# Wenshen-Jianpi prescription, a Chinese herbal medicine, improves visceral hypersensitivity in a rat model of IBS-D by regulating the MEK/ERK signal pathway

**DOI:** 10.3389/fphar.2022.955421

**Published:** 2022-09-23

**Authors:** Tianyuan Jiang, Ran Niu, Qian Liu, Yuhan Fu, Xiaoying Luo, Tao Zhang, Baoqi Wu, Juan Han, Yang Yang, Xiaolan Su, Jiande D. Z. Chen, Gengqing Song, Wei Wei

**Affiliations:** ^1^ Wangjing Hospital, China Academy of Chinese Medical Sciences, Beijing, China; ^2^ Laboratory of Functional Gastrointestinal Disorders Diagnosis and Treatment of Traditional Chinese Medicine, Beijing, China; ^3^ Department of Internal Medicine, MetroHealth Medical Center/Case Western Reserve University, Cleveland, OH, United States; ^4^ Institute of Acupuncture and Moxibustion, China Academy of Chinese Medical Sciences, Beijing, China; ^5^ Division of Gastroenterology and Hepatology, Department of Internal Medicine, University of Michigan, Ann Arbor, MI, United States; ^6^ Department of Gastroenterology and Hepatology, MetroHealth Medical Center/Case Western Reserve University, Cleveland, OH, United States

**Keywords:** rat model of IBS-D, visceral hypersensitivity, Chinese herbal medicine, colon, hippocampus, MEK/ERK signal pathway

## Abstract

The goal of the study was to analyze whether WJP can alleviate visceral hypersensitivity in IBS-D model rats. In this study, 36 Sprague–Dawley (SD) rats aged 4 weeks old were randomly divided into two groups: the model group (*n* = 27) and the control group (*n* = 9). The rat model of IBS-D was established by modified compound methods for 4 weeks. After the modification, IBS-D rats were randomly divided into three groups, namely, the IBS-D model group (*n* = 9), the positive drug group (*n* = 9), and the WJP group (*n* = 9), with different interventions, respectively. The control group was fed and allowed to drink water routinely. The Bristol stool scale scores were used to assess the severity of diarrhea. Abdominal withdrawal reflex (AWR) scores were used to assess visceral sensitivity. Expression of TNF-α was measured, and histopathological examinations were performed to assess colon inflammation in IBS-D model rats. Key factors of the MEK/ERK signal pathway in the tissue of the colon and hippocampus were measured to analyze the mechanism of WJP. Compared with the control group, the Bristol stool scale scores in the model group were significantly increased (*p* < 0.0001). The scores of the WJP group were significantly decreased compared with the model group (*p* = 0.0001). Compared with the control group, AWR scores in the model group at each pressure level were significantly increased (*p* = 0.0003, *p* < 0.0001, *p* = 0.0007, and *p* = 0.0009). AWR scores of the WJP group were significantly decreased compared with the model group (*p* = 0.0003, *p* = 0.0007, *p* = 0.0007, and *p* = 0.0009). Compared with the control group, the model group had significantly higher expression of TNF-α in the colon tissue (*p* < 0.0001). However, the WJP group had significantly lower level of TNF-α compared with the model group (*p* < 0.0001). Meanwhile, compared with the control group, the relative expression of the proteins of p-MEK1/2, p-ERK1, and p-ERK2 in the colon tissue was significantly increased in the model group (*p* < 0.0001). Compared with the model group, the relative expression of the proteins in the colon tissue were significantly decreased in the WJP group (*p* < 0.0001, *p* = 0.0019, and *p* = 0.0013). Compared with the control group, the relative expression of the proteins of p-MEK1/2, p-ERK1, and p-ERK2 in the hippocampus tissue were significantly increased in the model group (*p* < 0.0001). Compared with the model group, the relative expression of the proteins in the hippocampus tissue were significantly decreased in the WJP group (*p* = 0.0126, *p* = 0.0291, and *p* = 0.0145). The results indicated that WJP can alleviate visceral hypersensitivity in IBS-D model rats, possibly mediated by downregulating the expression of TNF-α, p-MEK1/2, p-ERK1, and p-ERK2 in the colon tissue. At the same time, WJP also affects downregulating the expression of p-MEK1/2, p-ERK1, and p-ERK2 in the hippocampus tissue.

## 1 Introduction

Irritable bowel syndrome (IBS) is characterized by recurrent abdominal pain associated with disordered defecation (Drossman et al.,2018). IBS can be further classified into four categories based on the predominant stool pattern by the Bristol Stool Form Scale: IBS with constipation (IBS-C), IBS with diarrhea (IBS-D), IBS with mixed stool pattern (IBS-M), and IBS unclassified (IBS-U). IBS-D was the most common subtype based on the Rome IV criteria [reported by 31.5% (95% CI 23.2–40.5; *I* ^2^ = 98.1% 61.6%) of people with IBS, corresponding to 1.4% (0.9%–1.9%) of all included participants having IBS-D] (Oka et al.,2020). The prevalence of IBS is between 5% and 10% in most geographical regions (Ford et al.,2020) and affects patients’ quality of life and increases socioeconomic burden (Black et al.,2020). The mechanisms that underlie the disease are complex, and it has a tendency of recurrence, making the development of an effective and safe treatment still a challenging task.

Wenshen-Jianpi prescription (WJP) is a Chinese herbal medicine. It has been shown to be an effective treatment option for IBS-D by alleviating abdominal pain and improving disordered defecation with a good safety profile (Su et al.,2013). Moreover, the results of our previous clinical trials also showed that WJP could decrease the recurrence rates in the treatment group compared to the control group (15.79% vs. 56.86%, *p* < 0.01). However, the therapeutic mechanism of WJP remains unclear.

Visceral hypersensitivity has been proposed to be one of the major pathophysiological mechanisms of IBS. Intestinal inflammation can be seen as one of the triggers, causing visceral hypersensitivity. TNF-α is the main proinflammatory cytokine, and IBS-D patients showed higher levels of TNF-αcompared with healthy controls (Russo et al.,2018). The mitogen-activated extracellular signal–regulated kinase (MEK)/extracellular regulated protein kinases (ERK) signaling pathway plays an important role in the formation and maintenance of visceral hypersensitivity (Sun et al.,2019). The MEK/ERK signaling pathway was recognized as a classical signal pathway with conservative evolution, regulating the basic cellular processes, including proliferation, differentiation, apoptosis, and metabolism, (O’Neill et al.,2004). MEK1/2 usually combines with the nonactivated form of p-ERK1/2, keeping ERK inside the cytoplasm (Wortzel et al.,2011). When MEK1/2 is activated, it can activate p-ERK1/2 by phosphorylating. Once p-ERK1/2 is activated, it detaches from MEK and goes into the nucleus, activating the downstream mediators, such as c-fos (Roskoski,2012), and magnifying the nociceptive stimulation to produce the manifestation of visceral hypersensitivity. Suppressing the expression of ERK can significantly relieve pain perception (Xu et al.,2008).

However, studies in animal post-inflammation models indicated that visceral hypersensitivity to mechanical or chemical stimuli persists after the inflammation has resolved (Coldwell JR et al.,2007; Lyubashina et al.,2022), and it inspired us to explore where the “memory” of the visceral afferent hyperexcitability is stored. Hippocampus is a key structure for cognition and memory. A previous study indicated that it plays an important role in interoceptive signaling driven by memory mechanisms. In addition, the disorders responses to the interoceptive signaling can induce clinically relevant phenotypes, including chronic visceral hypersensitivity (Labrenz et al.,2022). At the same time, adverse life events, stress, and anxiety/depression can aggravate the degree of abdominal pain (Lyubashina et al.,2022).

Thus, the goal of this study was to explore the therapeutic mechanism of WJP in treating IBS-D model rats and to determine whether it can alleviate visceral hypersensitivity *via* the MEK/ERK signaling pathway in the region of the colon and hippocampus.

## 2 Experimental materials and methods

### 2.1 Chemicals and reagents

ELISA Kit for tumor necrosis factor-alpha (TNFa) (BIO EXCELLENCE, SEA133Ra), Kit for TRNzol Universal Reagent (TIANGEN BIOTECH (BEIJING) CO., LTD., DP424), Kit for EasyScrpt One-Step gDNA Removal and cDNA Synthesis SuperMix (TransGen Biotech, AE311-03), Kit for Hieff™ qPCR SYBR^®^ Green Master Mix (No Rox), (Shanghai Yeasen BioTechnologies Co., Ltd., 11201ES08), MEK1/2 antibody (Affinity Biosciences, AF6385), Phospho-MEK1/2 (Ser218 + Ser222/Ser222 + Ser226) antibody (Affinity Biosciences, AF8035), p-ERK1/2 Polyclonal antibody (Cell Signaling Technology, 4695s), and Phospho-ERK1/2 (Thr202/Tyr204) Polyclonal antibody (Cell Signaling Technology, 4695s) were used in this study. Primers to amplify the genes GAPDH, MEK1, MEK2, ERK1, and ERK2 were designed by GENERAL BIOL (Anhui) Co., Ltd. (Anhui, China) (Table 1). Senna leaf and WJP formula granules (Sichuan Neo-Green Pharmaceutical Technology Development Co., Ltd., China) and pinaverium bromide (MYLAN LABORATORIES SAS, France) were used in this study.

**TABLE 1:** Constituents of WJP.

Chinese Name	Botanical Name	Genus	Family	Weight (g)	Part Used
Bu Gu Zhi	Cullen corylifolium	Psoralea Linn.	Fabaceae	30	Fruit
Rou Dou Kou	Myristica fragrans	Myristica Gronov.	Myristicaceae	15	Stem
Wu Zhu Yu	Tetradium ruticarpum	Evodia J. R. et G. Forst.	Rutaceae	6	Fruit
Wu Wei Zi	Schisandra chinensis	Schisandra Michx.	Schisandraceae	9	Fruit
Tai Zi Shen	Radix pseudostellariae	Pseudostellaria Pax	Caryophyllaceae	30	Root
Yu Jin	Curcuma aromatica	Curcuma L.	Zingiberaceae	18	Root
Chao Bai Zhu	Atractylodes macrocephala	Atractylodes	Asteraceae	10	Rhizome
Sheng Jiang	Zingiber officinale Roscoe	Zingiber	Suberitidae	10	Rhizome
Hong Zao	Ziziphus jujuba Mill.	Ziziphus Mill.	Rhamnaceae	10	Fruit

Abbreviation: WJP, Wenshen-Jianpi prescription.

### 2.2 Preparation and high-performance liquid chromatography analysis of WJP

**FIGURE 1:**
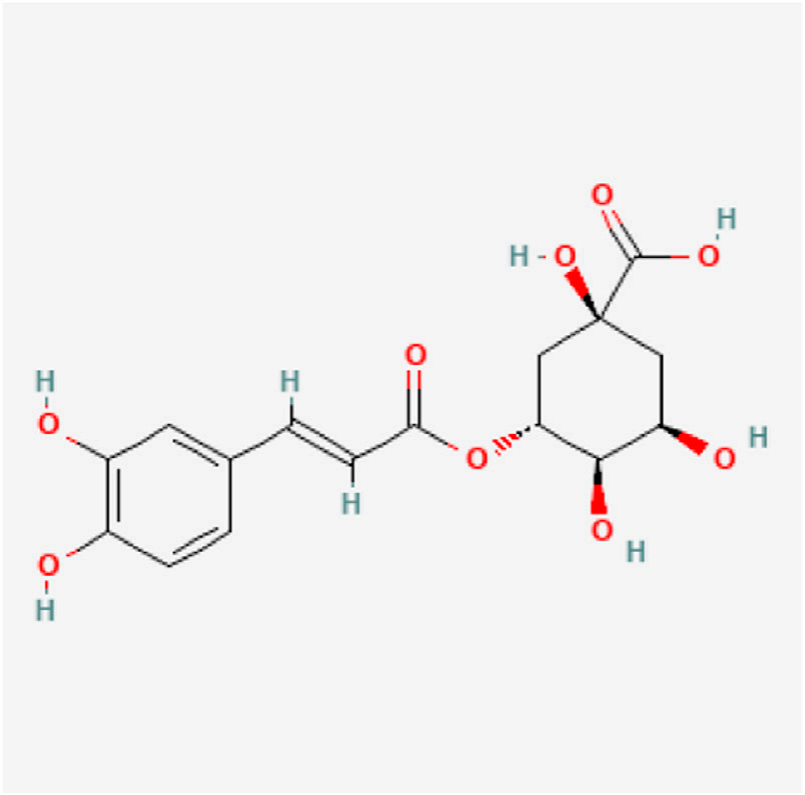
Chemical construction of Caffeoyl quinic acid.

WJP was prepared from nine commonly used herbs (Table 1), purchased from Beijing Sifang traditional Chinese medicine decoction pieces Co., Ltd. The nine ingredients were mixed according to the ratio of 30: 15: 6: 9: 30: 18: 10: 10: 10 (dry weight in grams). All herbs were decocted twice. The decoction of traditional Chinese Medicine was filtered using a 0.22 µm filter membrane, and 10 μl of the supernatant was collected for the test. Caffeoyl quinic acid (isomer of 831, 833, 834) (the representative component of WJP) was determined by HPLC, the Figure 1 showed the molecular structures (https://pubchem.ncbi.nlm.nih.gov/compound/348159). And 83 compounds were detected (the data have not been published).

### 2.3 Ethics statement

All feeding conditions were in compliance with the Chinese Animal Welfare Law and the relevant regulations of the Chinese Academy of Chinese Medical Sciences Experimental Animal Ethics Committee. Sprague–Dawley (SD) rats from the SiPeiFu (Beijing) Biotechnology Co., Ltd. (Beijing, China) were recruited to establish the model of IBS-D. Animal license Code: SCXK Beijing 2019-0010. Ethics No.: D2021-03-16-3.

### 2.4 Experimental protocol

**FIGURE 2:**
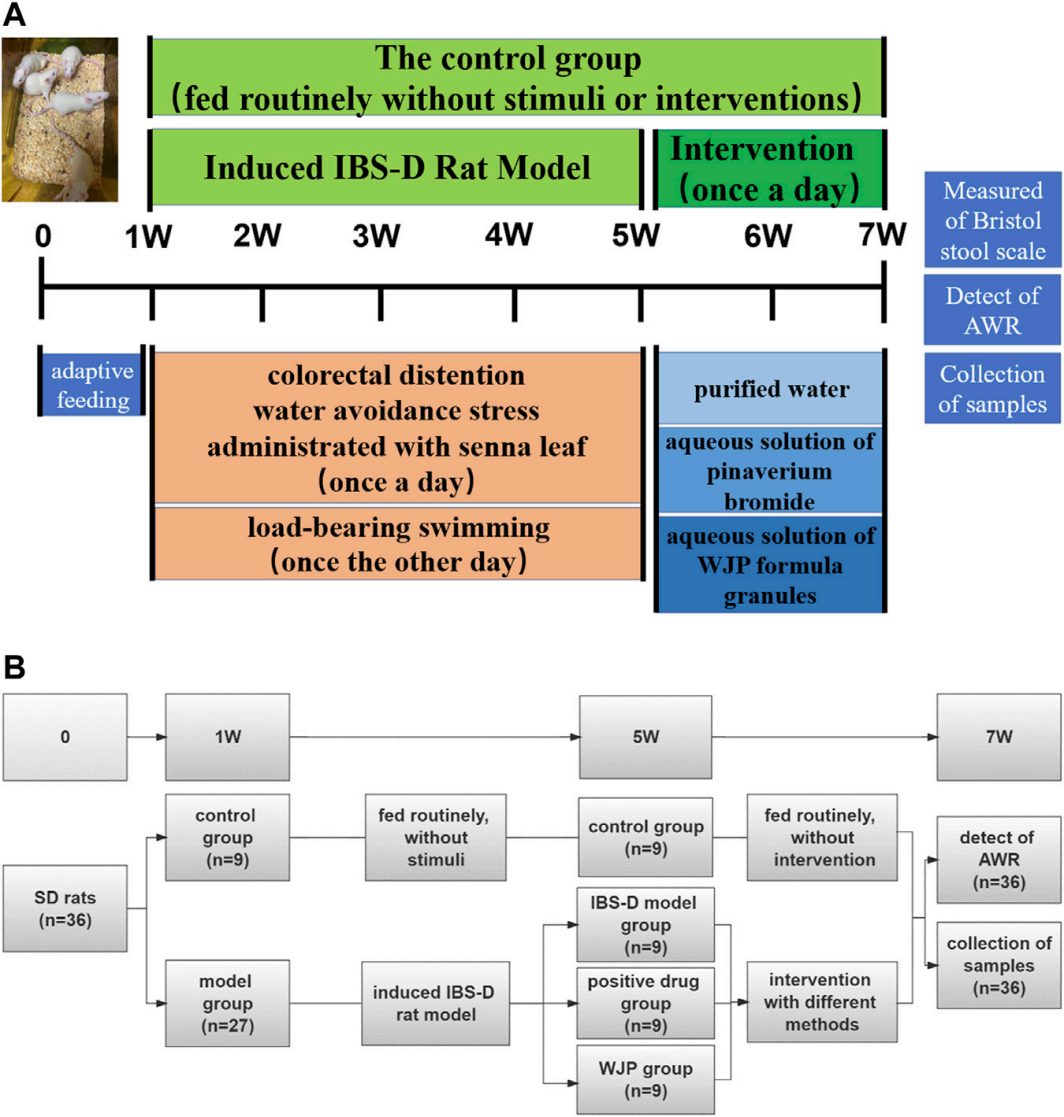
Detailed procedures for the experiments and their general conditions. **(A)** Protocol diagram of the time course involved in the experimental procedures of the IBS-D rat model induced and the intervention. **(B)** Specific numbers of animals in each group of the experiment.

A total of 36 male Sprague–Dawley (SD) rats (aged 4 weeks old and weighing 84.11 ± 10.02) were purchased from the SiPeiFu (Beijing) Biotechnology Co., Ltd. (Beijing, China). The rats were housed in an animal room (23 ± 2°C, 60% ± 5% relative humidity) with the setting of a 12 h dark/12 h light cycle. Before the experiment, the rats were given water and fed standard laboratory food for acclimatization for a week. The experiment was divided into two phases. And Figure 2 is a schematic diagram showed the detailed procedures for the experiments and the general conditions.

#### 2.4.1 IBS-D rat model preparation

Rats were randomly divided into two groups: the control group (*n* = 9) and the model group (*n* = 27). The control group was fed routinely and only received routine tests but not stimuli or therapies. Meanwhile, the model group was stimulated according to the modified compound methods, including colorectal distention (Al-Chaer et al.,2000), water avoidance stress (Bradesi et al.,2005), load-bearing swimming, and administration of the senna leaf gavage (Li et al.,2017a). Four weeks later, the rats in the model group were randomly divided into the IBS-D model group (*n* = 9), the positive drug group (*n* = 9), and the WJP group (*n* = 9).

To be more specific,

a. Colorectal distention (CRD):

The distention was applied using an apparatus, that is, a latex balloon (length, 20.0 mm; diameter, 3.0 mm) attached to a sphygmomanometer. The balloon was inserted rectally into the descending colon, exerting a pressure of 60 mmHg (as measured with a sphygmomanometer) for 1 min, and then it was deflated and withdrawn. The distention was repeated two times (separated by 30 min) per hour daily for 4 weeks.

b. Water avoidance stress (WAS):

The apparatus consisted of a plastic tank (55 cm length × 35 cm width × 35 cm height) with a block (10 × 8 × 8 cm) affixed to the center of the floor. The tank was filled with fresh room temperature water (25 ± 2)°C to within 1 cm of the top of the block. The rats were placed on the block for the duration of 1 h daily for 4 weeks.

c. Load-bearing swimming:

The apparatus was a plastic bucket (60 cm height, 40 cm diameter). The bucket was filled with fresh room temperature water (25 ± 2)°C with a depth of about 50 cm. The rats were made to swim in the apparatus till they were exhausted, with an iron block of 10% of body weight attached to the tail. The criterion to judge the exhaustion of rats was that their heads did not surface within 7 s after sinking into the water. This process was repeated once every other day for 4 weeks.

d. Administration with Senna leaf:

Senna leaf formula granules dissolved in purified water with a ratio of 1:7, 10 ml/(kg d) were administrated daily for 4 weeks.

#### 2.4.2 Intervention in different groups

The WJP group were administered an aqueous solution of WJP with 0.11 g/ml (WJP formula granules dissolved in purified water), 10 ml/(kg d) (the dosage for the rat = X mg/kg × 70 kg × 0. 018/200 g. X = 12.20 g/70 kg, the dosage for the adult daily). The positive drug group was given an aqueous solution of pinaverium bromide with 2.70 mg/ml (pinaverium bromide tablet dissolved in purified water), 10 ml/(kg d) (the dosage for the rat = X mg/kg × 70 kg × 0. 018/200 g. X = 150 mg/70 kg, the dosage for the adult daily). Meanwhile, the model group was given purified water, 10 ml/(kg d), administered *via* oral gavage daily for 2 weeks. After the assessment of diarrhea and visceral sensitivity at the end of the 7th week, all rats were sacrificed for samples.

### 2.5 Bristol stool scale score and abdominal withdrawal reflex score

Bristol stool scale was used to measure the symptom of diarrhea. The rats were placed inside a cage mat with filter paper, observed within 4 h (8:00–12:00) of defecation, and the Bristol stool scale scores were recorded according to the Bristol stool scale (Lewis et al.,1997).

**FIGURE 3:**
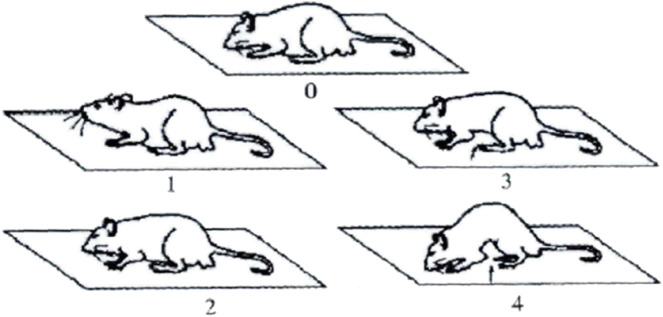
AWR diagram.

Abdominal withdrawal reflex (AWR) was used to measure the visceral sensitivity of rats in each group. The rats were starved for 18 h before the measurement. They were placed on a laboratory table matted with a single-use medical pad sheet. One of the operators, wearing thick gloves, held the rat gently and covered its eyes to restrain it from big movements like turning around. The other operator performed the operation of inserting the balloon. At the beginning of the experiment, it is necessary to touch the anus gently to achieve muscular relaxation. A balloon coated with paraffin oil was inserted 7 cm into the rectum *via* the anus and held in place by taping the tubing to the tail. Then, the rat was placed in a small transparent plastic cubicle (20 cm × 8 cm × 8 cm) (Winston et al.,2007) to restrain it from big movements like turning around. After the rat was completely calm, the balloon was slowly inflated to a pressure of 20, 40, 60, and 80 mmHg incrementally at a duration of 20 s each. AWR was scored as follows (Zhang et al.,2016): 0, no behavioral response to distension; 1, brief head movement followed by immobility; 2, contraction of the abdominal muscle; 3, lifting of the abdomen; or 4, body arching and lifting of the pelvic structure (Figure 3) (Jiajie Zhu,2018). Two blinded investigators conducted the experiments and assessed the scores of the Bristol stool scale and AWR.

### 2.6 ELISA of the colon tissue

The tissue of the colon was homogenized, mixed with PBS solution (10 ml PBS for 1 g of tissue), and centrifuged at 4,500 rpm for 5 min to obtain the supernatant. The concentrations of TNF-α were measured by ELISA according to the kit instructions.

### 2.7 Colon histopathology

The colon of rats in each group (3 cm of the colon 5–8 cm distal to the anus) was intercepted, washed with normal saline, and fixed with 10% neutral formalin solution for standby. Paraffin specimens were continuously sectioned at a thickness of 2 μm and stained with hematoxylin–eosin, and the histopathological manifestations of the colon in each group were visualized using Case Viewer 2.1 software.

### 2.8 Real-time polymerase chain reaction

Total RNA was extracted from tissue samples of the colon and hippocampus using the method of Trizol. The concentration and purity of the sample were determined using a spectrophotometer. The total RNA was then reverse transcribed into cDNA using the EasyScrpt One-Step gDNA Removal and cDNA Synthesis SuperMix kit. PCR primers were designed and synthesized by GENERAL BIOL. The primer sequences are shown in the Table 2. Finally, real-time PCR was performed on target genes, and the data were analyzed using the 2^−ΔΔCt^ method.

**TABLE 2:** Sequence of gene primers.

Gene official symbol	Forward primer sequence	Reverse primer sequence	Product length (bp)
GAPDH	CAA​GTT​CAA​CGG​CAC​AGT​CAA​G	ACA​TAC​TCA​GCA​CCA​GCA​TCA​C	123
MEK1	AAG​AAG​AAG​CCG​ACG​CCC​A	CCT​TCA​ACT​CTC​CCA​CCT​TCT​G	187
MEK2	CAT​CAG​TGC​CAC​CTC​CCA​AG	TGA​AGG​CAT​GGT​TCG​TCA​GC	129
ERK1	ACC​TGC​TGG​ACC​GGA​TGT​TA	AGC​CAC​TGG​TTC​ATC​TGT​CG	113
ERK2	CAC​CCG​CAT​TGG​TAG​GAA​CT	GGA​TTG​ATC​TGC​GGT​TGT​GC	167

### 2.9 Western blot analysis

The colon and hippocampus tissues were cut into small pieces and mixed with pre-cooled lysis buffer at a ratio of 1:20, respectively. The samples were centrifuged at 10,000 rpm for 5 min to obtain the supernatant. The protein concentration of each sample was determined according to the kit’s instructions. 10 μl of the supernatant was separated by 12% SDS-PAGE for 1 h at 150 V. Bands were electrophoretically transferred to 0.22 μm PVDF membranes (Millipore, United States), blocked for nonspecific binding with 5% nonfat dry milk for 1 h, shaken, and washed for 3 min twice. Then, the membranes were incubated with Linked Caprine Anti-Rabbit IgG Polyclonal Antibody and Linked Caprine Anti-Mouse IgG Polyclonal Antibody (Cloud-Clone, China) for 1 h, washed again, and developed with an electrochemiluminescence (ECL) reagent (Yeasen, China). Blots were developed *via* autoradiography. GAPDH (Cloud-Clone, China, 0.03 μg/ml) served as an internal control. Images were analyzed and quantified using ImageJ software. The following primary antibodies were used: MEK1/2 antibody (Affinity Biosciences, United States, 1:1,000), Phospho-MEK1/2 (Ser218 + Ser222/Ser222 + Ser226) antibody (Affinity Biosciences, United States, 1:1,000), p-ERK1/2 Polyclonal antibody (Cell Signaling Technology, United States, 1:1,000), Phospho-ERK1/2 (Thr202/Tyr204) Polyclonal antibody (Cell Signaling Technology, United States, 1:1,000).

### 2.10 Statistical analysis

Statistical analyses were performed using Graphpad Prism software version 9.2.0. Experiments were repeated at least three times, and results were presented as mean ± standard deviation (SD). One-way ANOVA was used to compare the significance of the differences among groups. The Brown–Forsythe and Welch ANOVA tests were applied when the variance was equal among groups, and the Kruskal–Wallis test was performed otherwise. Spearman rank correlation analysis was used to analyze the correlation of data with non-normality and described by correlation coefficient. *p* < 0.05 was considered statistically significant.

## 3 Results

### 3.1 WJP ameliorated the symptom of diarrhea of IBS-D rats

**FIGURE 4:**
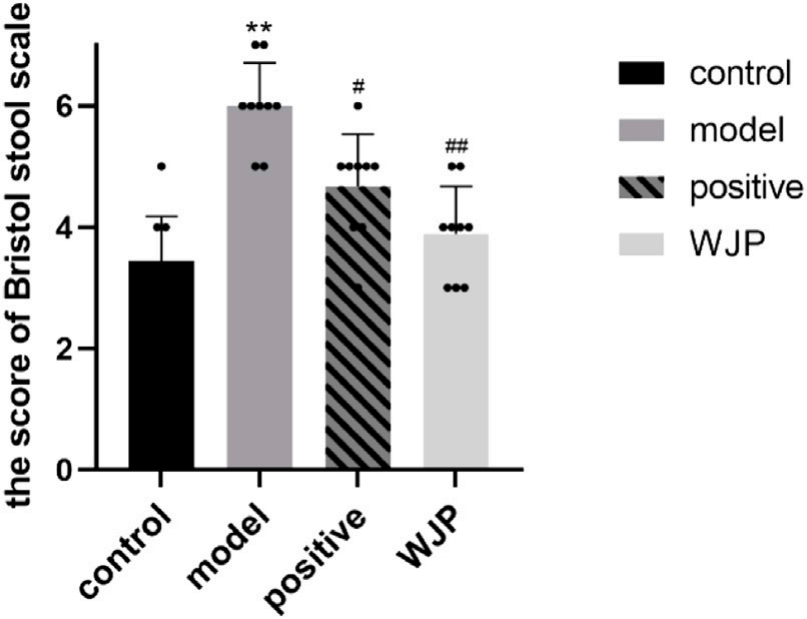
Comparison of Bristol stool scale score.

Bristol stool scale score was measured to determine whether Wenshen-Jianpi prescription could ameliorate the symptom of diarrhea of IBS-D rats (Figure 4). The Bristol stool scale score of the model group was significantly increased compared with the control group (*p* < 0.0001). It suggested that the IBS-D rats had a variety of fecal patterns, which is consistent with the clinical manifestations of IBS-D patients. Compared with the model group, the Bristol stool scale score of the WJP group was significantly decreased (*p* = 0.0001). These results indicated that Wenshen-Jianpi prescription ameliorated diarrhea in IBS-D models.

### 3.2 WJP ameliorated visceral hypersensitivity of IBS-D rats

**FIGURE 5:**
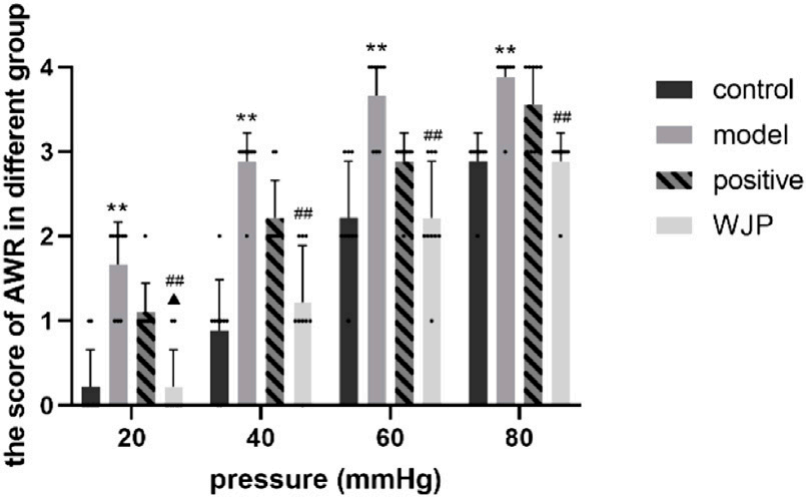
Comparison of AWR score. *p < 0.05; **p < 0.01, versus the control group. ^#^ *p* < 0*.*05; ^##^ *p* < 0*.*01, versus the model group.

The AWR scores were measured to determine whether Wenshen-Jianpi prescription could ameliorate visceral hypersensitivity of IBS-D rats (Figure 5). Our result showed that the AWR score of the model group increased significantly compared with the control group at each pressure level (20 mmHg, 40 mmHg, 60 mmHg, 80 mmHg) (*p* = 0.0003, *p* < 0.0001, *p* = 0.0007, and *p* = 0.0009). It suggested that the IBS-D rats had visceral hypersensitivity, which is consistent with the clinical manifestations of IBS-D patients. The AWR scores of WJP group were significantly decreased compared with the model group at each pressure level (*p* = 0.0003, *p* = 0.0007, *p* = 0.0007, and *p* = 0.0009). Compared with positive drug group, the AWR scores of WJP group were significantly decreased at the pressure level of 20 mmHg (*p* = 0.0403). These results indicated that Wenshen-Jianpi prescription effectivity ameliorated the visceral hypersensitivity of IBS-D rats and is superior to the positive drug group at the pressure at 20 mmHg. These results indicated that Wenshen-Jianpi prescription ameliorated visceral hypersensitivity in IBS-D models.

### 3.3 WJP ameliorated the inflammation of IBS-D rats

**FIGURE 6:**
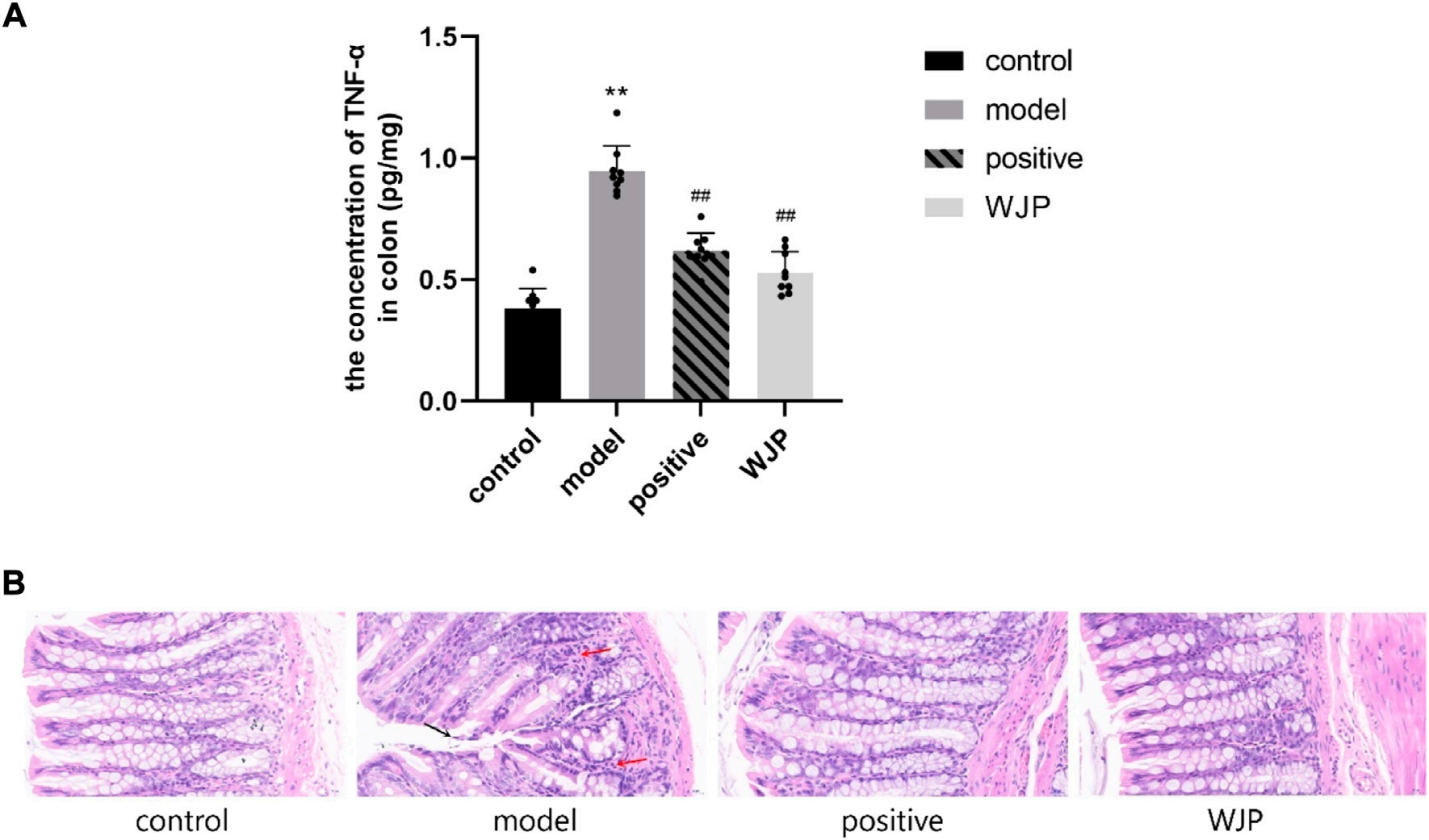
Comparison of the concentration of TNF-α in the colon and the histopathological observation of colon tissue. **(A)** Concentration of TNF-α. *p < 0.05; **p < 0.01, versus the control group. ^#^ *p* < 0.05; ^##^ *p* < 0.01, versus the model group. **(B)** Histological changes in each group observed after HE staining. Magnification ×400. A small amount of epithelial cell shedding can be seen in local mucosa (black arrow). More lymphocyte infiltration can be seen in local lamina propria (red arrow).

To determine whether Wenshen-Jianpi prescription could alleviate the inflammation of IBS-D rats, the level of TNF-α in the colon tissues was detected using ELISA, and the colon tissues were stained with HE stain. Compared with the control group, the concentration of TNF-α was significantly increased in the model group (*p* < 0.0001). Compared with the model group, the level of TNF-α in the WJP group was significantly decreased (*p* < 0.0001). Even though there was no significant difference between the positive drug group and the WJP group (*p* > 0.05), the results showed the consistent trend that the level of TNF-α was lower in the WJP group (*p* = 0.151, 0.62 ± 0.072 vs. 0.53 ± 0.086) (Figure 6A).

Immunohistochemistry staining showed that intestinal mucosa and the epithelial cells were intact in the control group without lymphocyte infiltration in the lamina propria. However, there was a small amount of epithelial cell shedding in the local mucosa in the model group (Figure 6B, black arrow) and more lymphocyte infiltration in local lamina propria (Figure 6B, red arrow).

### 3.4 WJP downregulated the expression of pMEK1/2, pERK1, and pERK2 to ameliorate the visceral hypersensitivity of IBS-D rats

**FIGURE 7:**
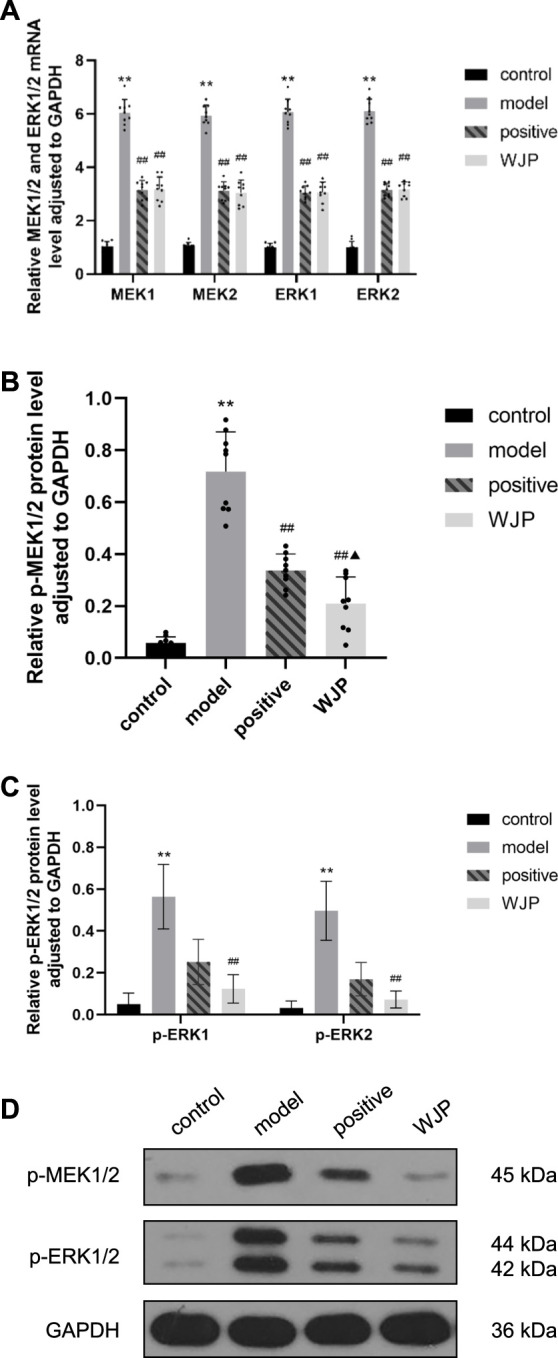
Effect of WJP on the relative expression of mRNA of MEK1, MEK2, ERK1, and ERK2 and the relative expression of protein of p-MEK1/2, p-ERK1, and p-ERK2 in the colon in the four groups. **(A)** The relative expression of mRNA of MEK1, MEK2, ERK1, and ERK2. **(B)** The relative expression of protein of p-MEK1/2. **(C)** The relative expression of protein of p-ERK1 and p-ERK2. **(D)** Western blot analysis. *p < 0.05; **p < 0.01, versus the control group. ^#^ *p* < 0.05; ^##^ *p* < 0.01, versus the model group. ^▲^ *p* < 0.05; ^▲▲^ *p* < 0.01, versus the positive drug group.

The MEK/ERK signaling pathway was also evaluated in the colon tissues. The mRNA levels of MEK1, MEK2, ERK1, and ERK2 in the colon tissues were significantly increased in the model group compared to the control group (*p* < 0.0001); however, those levels were significantly decreased in the WJP group when compared with the model group (*p* < 0.0001). There was no significant difference between the positive drug group and the WJP group (*p* > 0.05) (Figure 7A).

Compared with the control group, the levels of p-MEK1/2 proteins of the colon tissues was significantly increased in the model group (*p* < 0.0001). Compared with model group, this levels was significantly decreased in the WJP group (*p* < 0.0001). Compared with the positive drug group, this levels was significantly decreased in the WJP group (*p* = 0.0390) (Figure 7B).

Compared with the control group, the relative expressions of p-ERK1 and p-ERK2 proteins in the colon tissues were significantly increased in the model group (*p* < 0.0001); whereas, those expressions were significantly decreased in the WJP group when compared with the model group (*p* = 0.0019 and *p* = 0.0013). (Figure 7C). The result of Western blot analysis is shown in Figure 7D.

**FIGURE 8:**
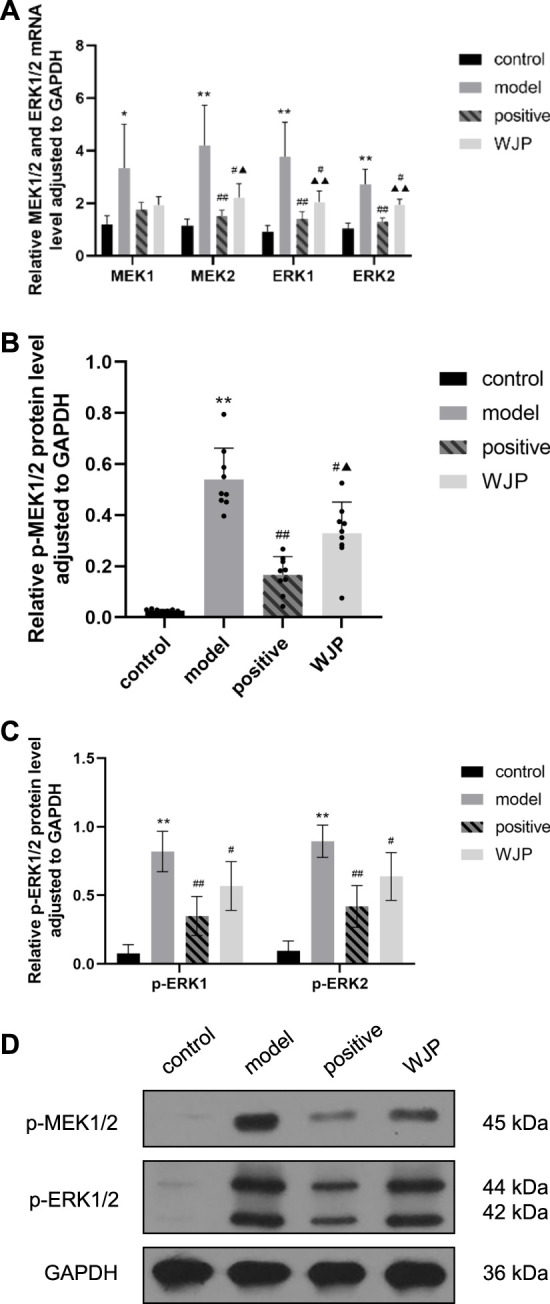
Effect of WJP on the relative expression of mRNA of MEK1, MEK2, ERK1, and ERK2 and the relative expression of protein of p-MEK1/2, p-ERK1, and p-ERK2 in the hippocampus in the four groups. **(A)** The relative expression of mRNA of MEK1, MEK2, ERK1, and ERK2. **(B)** The relative expression of protein of p-MEK1/2. **(C)** The relative expression of protein of p-ERK1 and p-ERK2. **(D)** Western blot analysis. *p < 0.05; **p < 0.01, versus the control group. ^#^ *p* < 0.05; ^##^ *p* < 0.01, versus the model group. ^▲^ *p* < 0.05; ^▲▲^ *p* < 0.01, versus the positive drug group.

The MEK/ERK signaling pathway was also evaluated in the hippocampus tissue. The mRNA levels of MEK1, MEK2, ERK1, and ERK2 were significantly increased in the model group compared with the control group (*p* = 0.0249, *p* = 0.0018, *p* = 0.0007, and *p* < 0.0001). However, the mRNA levels of MEK2, ERK1, and ERK2 were significantly decreased in the WJP group when compared with the model group (*p* = 0.0215, *p* = 0.0217, and *p* = 0.0189). Compared with the positive drug group, the relative mRNA levels of MEK2, ERK1, and ERK2 were significantly increased in the WJP group (*p* = 0.0198, *p* = 0.0089, and *p* < 0.0001) (Figure 8A).

The relative expression of p-MEK1/2 protein was significantly increased in the model group compared with the control group (*p* < 0.0001); the expression of p-MEK1/2 protein was significantly decreased in the WJP group compared with the model group (*p* = 0.0126). Compared with the positive drug group, the expression of p-MEK1/2 protein was significantly increased in the WJP group (*p* = 0.0240) (Figure 8B). Compared with the control group, the relative expressions of p-ERK1 and p-ERK2 proteins were increased in the model group (*p* < 0.0001). Compared with the model group, the relative expressions of p-ERK1 and p-ERK2 proteins were decreased in the WJP group (*p* = 0.0291 and *p* = 0.0145) (Figure 8C). The result of Western blot analysis is shown in Figure 8D.

### 3.5 The correlation between the abdominal withdrawal reflex score and the expression of p-p-ERK1/2 in the colon and hippocampus

**FIGURE 9:**
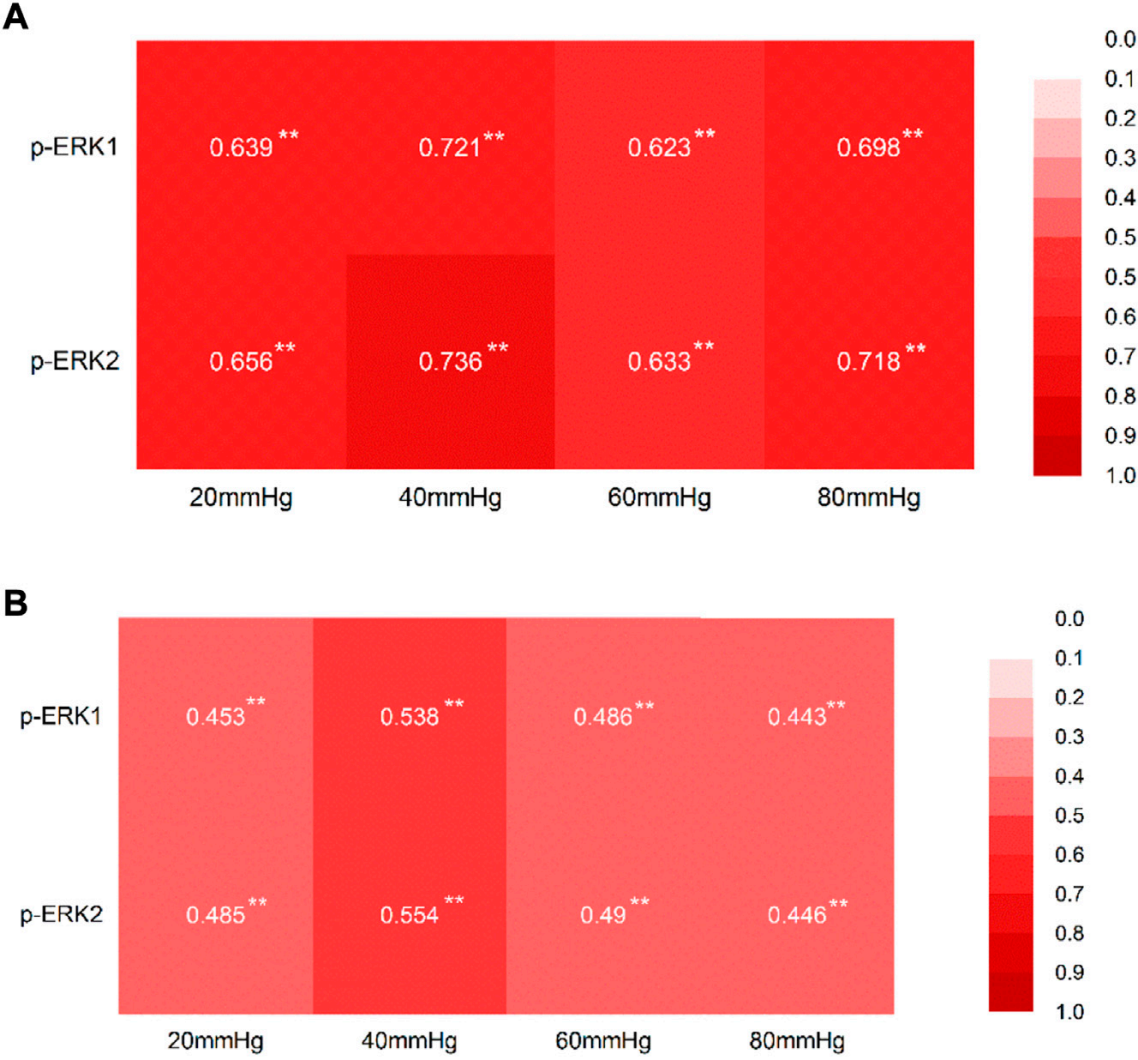
Correlation between the AWR score at each pressure level and the expression of p-ERK1/2. **(A)** Correlation between the AWR score at each pressure level and the expression of p-ERK1/2 in the tissue of the colon, with the correlation coefficient. **(B)** Correlation between the AWR score at each pressure level and the expression of p-ERK1/2 in the tissue of the hippocampus, with the correlation coefficient. *p < 0.05; **p < 0.01.

The AWR scores positively correlated with the expression of p-p-ERK1/2 in both colon tissues and the hippocampus tissues at each pressure level (Figures 9A and B).

## 4 Discussion

**FIGURE 10:**
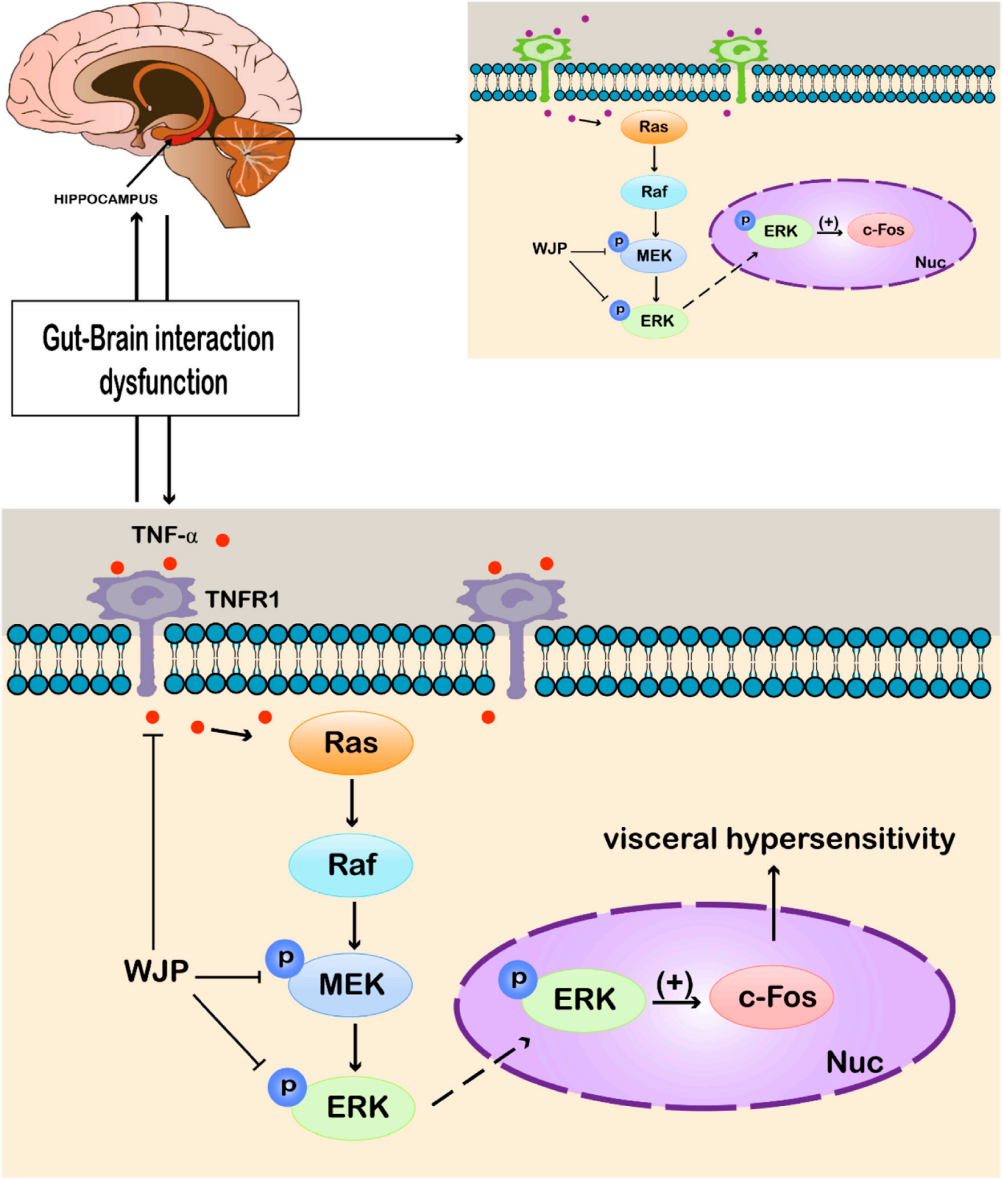
WJP regulation of the MEK/ERK signal pathway. Abbreviations: WJP, Wenshen-Jianpi Prescription; TNF-α, tumor necrosis factor-α; TNFR1, tumor necrosis factor receptor-1; MEK, mitogen-activated extracellular signal–regulated kinase; ERK, extracellular regulated protein kinases; p, phosphorylation; Nuc, cell nucleus; (+), activating the expression.

Our study has investigated the Bristol stool scale and AWR scores, inflammatory cytokine, histopathological observation, and the key factors of the MEK/ERK signal pathway among different groups. And Figure 10 is a schematic diagram showed the WJP regulation of the MEK/ERK signal pathway.

The Bristol stool scale was used to measure the symptoms of diarrhea, and AWR was used to evaluate the visceral sensitivity. The findings confirmed that the model rats developed diarrhea and had higher visceral sensitivity at each pressure level compared to the control group, which is consistent with the findings of Zhang et al. (2020). After intervention with the WJP, the Bristol stool scale scores were decreased, and the AWR scores were decreased compared to the model group at each pressure level.

TNF-α is a proinflammatory cytokine, the level of which was increased in IBS patients compared with the healthy controls (Norlin et al.,2021). Our study showed consistent findings that the level of TNF-α is increased in the IBS-D model group. In addition, histopathology staining showed that there was more lymphocyte infiltration in local lamina propria in the IBS-D model group compared with the control group.

In their study, Liu et al. (2018) indicated that the expressions of mRNA of MEK1, MEK2, ERK1, and ERK2 were increased in the spinal cord in rats’ model of visceral pain. Our study findings confirmed that these parameters were also increased in the colon and hippocampus in the rat model of IBS-D with visceral hypersensitivity compared to the control group. In addition, after the intervention with WJP, the expression of MEK1, MEK2, ERK1, and ERK2 decreased in the colon, and that of MEK2, ERK1, and ERK2 decreased in the hippocampus.

In their study, Li et al. (2017b) indicated that the expressions of proteins of p-MEK1/2 and p-p-ERK1/2 were increased in the spinal cord in rat’s model of visceral pain. Our study findings confirmed that the expressions of proteins of p-MEK1/2, p-ERK1, and p-ERK2 were increased in the colon tissue in the model group compared to the control group. In addition, the expressions of proteins of p-MEK1/2, p-ERK1, and p-ERK2 were also increased in the hippocampus tissue in the model group compared to the control group. The literature research indicated that these proteins are active after phosphorylation (Zhao et al.,2022) and can activate downstream proteins to produce biological effects. Thus, we considered p-MEK1/2, p-ERK1, and p-ERK2 as the key effect parameters and played a significant role in the development of visceral hypersensitivity.

Visceral hypersensitivity has been proposed to be the core pathophysiological mechanism behind IBS (Collaborative group of Gastrointestinal Functional Diseases of Gastroenterology Branch of Chinese Medical Association, 2020), leading to decreased pain threshold and changes in synaptic plasticity (SP) (Greenwood-Van et al., 2017). SP refers to the ability of the nervous system to reconstruct connections in order to adapt to the structural and functional changes in the environment. The changes in synaptic transmission function are mainly regulated by long-term potentiation (LTP) and long-term depression (LTD) (Bennett,2000). LTP strengthens specific synapses, while LTD weakens specific synapses, where LTP is crucial in the formation of visceral sensitization (Ruscheweyh et al.,2011). At the same time, MEK/ERK signaling pathway plays an important role in regulating LTP (Sun et al.,2015). Continuous inflammatory stimulation may activate the MEK/ERK signaling pathway. p-MEK1/2 can activate p-ERK1/2 by phosphorylation. p-ERK1/2 can activate the downstream factors involved in visceral sensitization, such as N-methyl-D-aspartic acid receptor (NMDAR) and c-fos; it also regulates the synaptic plasticity and excitability of cell membrane and amplifies peripheral stimuli (WU Hao-meng et al., 2020). Our previous results are consistent with this conclusion (Shi haixia et al.,2021; Jiang tianyuan et al.,2022). ERK is also the key factor of the mediator of the early-LTP and late-LTP, and the latter period is vital to the maintenance of LTP (Kelly et al.,2007). Visceral hypersensitivity along with the dysfunction of the “brain–gut axis” is thought to be contributing to the IBS-D symptoms (Morreale et al.,2022; Nozu et al.,2022). The “brain–gut axis” is a concept of neuroanatomy. It can be seen as the “connecting line” between the brain and the intestine, transforming the signals, from the emotional center to the cognitive center of the brain, through the neurotransmitters to regulate the function of the surrounding organs, including the intestine (Douglas A. DROSSMAN,2018), and vice versa, which is the biological basis of “gut-brain interaction.” Structurally, it is a bidirectional connection between the CNS and visceral smooth muscle and other peripheral organs, which affects the functions of sensation, movement, endocrine, autonomic nerve, immunity, and inflammation. The changes in synaptic plasticity may occur both in the periphery and central. The hippocampus is a key part of memory and cognition, and repeated stress can strengthen the neural connections related to traumatic stimulation in the hippocampus, leading to the persistence of hypersensitivity (Lyubashina et al.,2022b). Therefore, targeting both the colon and hippocampus may be a more effective approach to treat IBS-D and may even alter the natural history of the disease.

In addition, after the intervention with the WJP, the expressions of the proteins of p-MEK1/2, p-ERK1, and p-ERK2 were decreased in the colon and hippocampus compared to the model group. Previous studies suggest that ERK2 may play a more important role in the formation of visceral hypersensitivity (Alter et al.,2010; Xu et al.,2008), whereas in our study, both of the expressions of the p-ERK1 and p-ERK2 were increased in the model group, and decreased after intervention with the decoction of WJP. Thus, further experiments should be performed to research the different functions of p-ERK1 and p-ERK2 and find whether there is a balance between the parameters.

The correlation coefficients, about the AWR score and expression of p-ERK1 and p-ERK2, with a range of 0.44–0.76, indicate that there are correlations between the AWR score and the expression of the p-ERK1and p-ERK2.

There is evidence that p-ERK1/2 is implicated in synaptic plasticity, which is a key mechanism of the formation of visceral hypersensitivity (Zhang et al.,2018). ERK signaling cascade seems essential for neuronal transcription to control long-term memory (Medina et al.,2018), not only in the central never system (CNS) but also in the enteric nervous system (ENS) (Zhang et al.,2018). p-ERK1/2 is implicated in the formation of visceral hypersensitivity. Cold exposure can cause increased visceral pain responses to colorectal distension, accompanied by the activation of the p-ERK1/2 pathway and c-Fos expression in nodose neurons (Chen et al.,2019). Thus, the literature studies were consistent with our findings.

In summary, the MEK/ERK signaling pathway plays an important role in the formation and maintenance of the LTP. Furthermore, it is considered that LTP plays a key role in the formation and maintenance of visceral hypersensitivity. This study may provide another perspective to recognize the mechanism of chronic abdominal pain (one of the main symptoms of IBS-D) and even the recurrence of IBS-D. In addition, Wenshen-Jianpi prescription (WJP), the Chinese herbal medicine, may provide a possible method to promote the normalization of the expression of the MEK/ERK signaling pathway for the treatment of IBS-D. The MEK/ERK signaling pathway has been regulated by WJP both in the region of the colon and hippocampus at the same time. However, the mediators of the interaction between the colon and hippocampus are unclear, which can be explored in further experiments.

## 5 Conclusion

The Wenshen-Jianpi prescription can downregulate the expression of TNF-α to relieve intestinal inflammation. In addition, it can downregulate the expression of p-MEK1/2, p-ERK1, and p-ERK2 in the colon and hippocampus at the same time to alleviate visceral hypersensitivity in the IBS-D model rats.

## Data Availability

The original contributions presented in the study are included in the article/Supplementary Material; further inquiries can be directed to the corresponding authors.
