# Corrigendum: Wenshen-Jianpi prescription, a Chinese herbal medicine, improves visceral hypersensitivity in a rat model of IBS-D by regulating the MEK/ERK signal pathway

**DOI:** 10.3389/fphar.2022.1075874

**Published:** 2022-11-21

**Authors:** Tianyuan Jiang, Ran Niu, Qian Liu, Yuhan Fu, Xiaoying Luo, Tao Zhang, Baoqi Wu, Juan Han, Yang Yang, Xiaolan Su, Jiande D. Z. Chen, Gengqing Song, Wei Wei

**Affiliations:** ^1^ Wangjing Hospital, China Academy of Chinese Medical Sciences, Beijing, China; ^2^ Laboratory of Functional Gastrointestinal Disorders Diagnosis and Treatment of Traditional Chinese Medicine, Beijing, China; ^3^ Department of Internal Medicine, MetroHealth Medical Center/Case Western Reserve University, Cleveland, OH, United States; ^4^ Institute of Acupuncture and Moxibustion, China Academy of Chinese Medical Sciences, Beijing, China; ^5^ Division of Gastroenterology and Hepatology, Department of Internal Medicine, University of Michigan, Ann Arbor, MI, United States; ^6^ Department of Gastroenterology and Hepatology, MetroHealth Medical Center/Case Western Reserve University, Cleveland, OH, United States

**Keywords:** rat model of IBS-D, visceral hypersensitivity, Chinese herbal medicine, colon, hippocampus, MEK/ERK signal pathway

In the published article, there was an error in Figure 2A as published. The version that was published was an old pre-review version. The corrected Figure 2A appears below:

**FIGURE 2:**
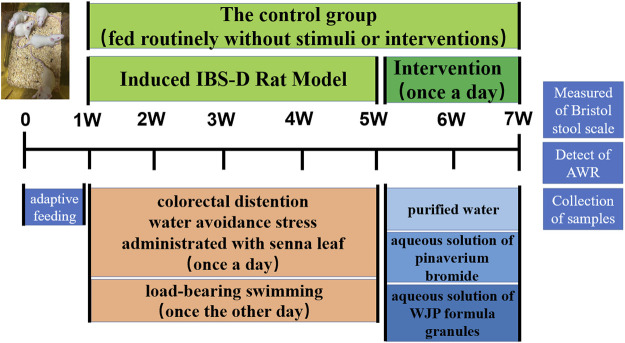
Detailed procedures for the experiments and their general conditions. **(A)** Protocol diagram of the time course involved in the experimental procedures of the IBS-D rat model induced and the intervention.

The authors apologize for this error and state that this does not change the scientific conclusions of the article in any way. The original article has been updated.

