# Supplementary material for: Wenshen-Jianpi prescription, a Chinese herbal medicine, improves visceral hypersensitivity in a rat model of IBS-D by regulating the MEK/ERK signal pathway
Source: Front Pharmacol. 2022 Sep 23;13:955421. doi: 10.3389/fphar.2022.955421 (PMC9540386; doi:10.3389/fphar.2022.955421)
Supplement: Supplementary file 1 [file DataSheet1.docx]

Supplementary Materials

1. the Bristol type score

Table 1 the Bristol type score (score)

| Group | Number | Bristol type | Bristol type score |
| --- | --- | --- | --- |
| Control group | 1 | 3 | 3 |
|  | 2 | 4 | 4 |
|  | 3 | 3 | 3 |
|  | 4 | 3 | 3 |
|  | 5 | 4 | 4 |
|  | 6 | 3 | 3 |
|  | 7 | 3 | 3 |
|  | 8 | 3 | 3 |
|  | 9 | 5 | 5 |
| Model group | 1 | 6 | 6 |
|  | 2 | 6 | 6 |
|  | 3 | 6 | 6 |
|  | 4 | 5 | 5 |
|  | 5 | 7 | 7 |
|  | 6 | 6 | 6 |
|  | 7 | 7 | 7 |
|  | 8 | 6 | 6 |
|  | 9 | 5 | 5 |
| Positive drug group | 1 | 5 | 5 |
|  | 2 | 5 | 5 |
|  | 3 | 5 | 5 |
|  | 4 | 4 | 4 |
|  | 5 | 3 | 3 |
|  | 6 | 5 | 5 |
|  | 7 | 6 | 6 |
|  | 8 | 4 | 4 |
|  | 9 | 5 | 5 |
| WJP group | 1 | 4 | 4 |
|  | 2 | 4 | 4 |
|  | 3 | 3 | 3 |
|  | 4 | 3 | 3 |
|  | 5 | 5 | 5 |
|  | 6 | 4 | 4 |
|  | 7 | 4 | 4 |
|  | 8 | 5 | 5 |
|  | 9 | 3 | 3 |

2. the AWR score at each pressure level and the Bristol type score in four groups

Table 2 The AWR score in four groups at each pressure level (score)

| Group | Number | Pressure level | | | |
| --- | --- | --- | --- | --- | --- |
|  |  | 20mmHg | 40mmHg | 60mmHg | 80mmHg |
| Control group | 1 | 0 | 1 | 3 | 3 |
|  | 2 | 0 | 1 | 2 | 3 |
|  | 3 | 0 | 1 | 2 | 3 |
|  | 4 | 0 | 0 | 2 | 3 |
|  | 5 | 0 | 0 | 1 | 2 |
|  | 6 | 1 | 2 | 3 | 3 |
|  | 7 | 1 | 1 | 2 | 3 |
|  | 8 | 0 | 1 | 3 | 3 |
|  | 9 | 0 | 1 | 2 | 3 |
| Model group | 1 | 2 | 3 | 4 | 4 |
|  | 2 | 2 | 3 | 4 | 4 |
|  | 3 | 2 | 3 | 4 | 4 |
|  | 4 | 2 | 3 | 3 | 4 |
|  | 5 | 2 | 3 | 4 | 4 |
|  | 6 | 1 | 2 | 3 | 3 |
|  | 7 | 2 | 3 | 4 | 4 |
|  | 8 | 1 | 3 | 4 | 4 |
|  | 9 | 1 | 3 | 3 | 4 |
| Positive drug group | 1 | 2 | 3 | 3 | 4 |
|  | 2 | 1 | 2 | 2 | 3 |
|  | 3 | 1 | 2 | 3 | 3 |
|  | 4 | 1 | 2 | 3 | 4 |
|  | 5 | 1 | 3 | 3 | 4 |
|  | 6 | 1 | 2 | 3 | 4 |
|  | 7 | 1 | 2 | 3 | 3 |
|  | 8 | 1 | 2 | 3 | 4 |
|  | 9 | 1 | 2 | 3 | 3 |
| WJP group | 1 | 0 | 1 | 2 | 2 |
|  | 2 | 1 | 2 | 3 | 3 |
|  | 3 | 0 | 1 | 2 | 3 |
|  | 4 | 0 | 2 | 3 | 3 |
|  | 5 | 0 | 1 | 2 | 3 |
|  | 6 | 1 | 2 | 3 | 3 |
|  | 7 | 0 | 1 | 2 | 3 |
|  | 8 | 0 | 1 | 2 | 3 |
|  | 9 | 0 | 0 | 1 | 3 |

3. the concentration of TNF-α in the colon tissue

Table 3 the concentration of TNF-*α* in the colon tissue (pg/mg)

| Group | Number | Final concentration |
| --- | --- | --- |
| Control group | 1 | 0.414467 |
|  | 2 | 0.53929 |
|  | 3 | 0.347118 |
|  | 4 | 0.414467 |
|  | 5 | 0.298952 |
|  | 6 | 0.318224 |
|  | 7 | 0.279672 |
|  | 8 | 0.433692 |
|  | 9 | 0.395234 |
| Model group | 1 | 0.892739 |
|  | 2 | 0.940296 |
|  | 3 | 0.864181 |
|  | 4 | 0.845133 |
|  | 5 | 0.921279 |
|  | 6 | 0.911768 |
|  | 7 | 0.949802 |
|  | 8 | 1.186804 |
|  | 9 | 1.016286 |
| Positive drug group | 1 | 0.58721 |
|  | 2 | 0.759317 |
|  | 3 | 0.654216 |
|  | 4 | 0.596788 |
|  | 5 | 0.625511 |
|  | 6 | 0.663781 |
|  | 7 | 0.596788 |
|  | 8 | 0.49132 |
|  | 9 | 0.596788 |
| WJP group | 1 | 0.635082 |
|  | 2 | 0.433692 |
|  | 3 | 0.606365 |
|  | 4 | 0.5297 |
|  | 5 | 0.663781 |
|  | 6 | 0.472119 |
|  | 7 | 0.443302 |
|  | 8 | 0.472119 |
|  | 9 | 0.510514 |

4. The relative expression of mRNA of MEK1, MEK2, ERK1, ERK2 and the relative expression of protein of MEK1/2, p-MEK1/2, ERK1, p-ERK1, ERK2, p-ERK2 in colon tissue in four groups.

Table 4 The relative expression of mRNA of MEK1, MEK2, ERK1, ERK2 in colon tissue

| Group | Number | MEK1 | MEK2 | ERK1 | ERK2 |
| --- | --- | --- | --- | --- | --- |
| Control group | 1 | 1.000000 | 1.000000 | 1.000000 | 1.000000 |
|  | 2 | 1.205808 | 1.32256 | 0.903335 | 1.307369 |
|  | 3 | 0.83702 | 1.127661 | 1.135504 | 0.788218 |
|  | 4 | 0.905425 | 1.059463 | 0.819794 | 0.922316 |
|  | 5 | 1.099362 | 1.074253 | 0.922316 | 0.83702 |
|  | 6 | 0.823591 | 1.047294 | 1.191958 | 1.417485 |
|  | 7 | 0.968171 | 1.042466 | 0.754364 | 0.82932 |
|  | 8 | 1.274561 | 0.886791 | 1.018656 | 0.97716 |
|  | 9 | 1.259921 | 1.057018 | 1.191958 | 0.972655 |
| Model group | 1 | 6.233317 | 5.883467 | 6.161721 | 5.829343 |
|  | 2 | 5.59188 | 6.048874 | 7.012846 | 6.393769 |
|  | 3 | 7.045327 | 5.683055 | 6.498019 | 5.979397 |
|  | 4 | 5.775717 | 5.762387 | 6.020987 | 5.842827 |
|  | 5 | 5.842827 | 5.278032 | 5.696201 | 6.603969 |
|  | 6 | 6.048874 | 6.543216 | 5.59188 | 5.669939 |
|  | 7 | 6.291192 | 5.669939 | 5.979397 | 6.090947 |
|  | 8 | 5.388934 | 6.305744 | 5.451551 | 5.61778 |
|  | 9 | 6.233317 | 6.276673 | 6.19026 | 6.932296 |
| Positive drug group | 1 | 2.560928 | 3.286761 | 3.450168 | 3.442206 |
|  | 2 | 3.418429 | 3.271608 | 3.024437 | 3.450168 |
|  | 3 | 3.630077 | 2.651239 | 2.874544 | 2.894538 |
|  | 4 | 3.256525 | 2.770219 | 3.301984 | 2.934945 |
|  | 5 | 3.363586 | 2.744735 | 3.24901 | 3.167475 |
|  | 6 | 3.426336 | 3.264058 | 3.10229 | 2.989698 |
|  | 7 | 2.757447 | 3.672256 | 2.79594 | 3.355823 |
|  | 8 | 3.052518 | 3.20428 | 2.682045 | 2.828427 |
|  | 9 | 2.802407 | 3.241511 | 3.059579 | 3.294364 |
| WJP group | 1 | 3.819366 | 3.348078 | 2.632925 | 3.317278 |
|  | 2 | 2.763826 | 2.519842 | 3.426336 | 2.815387 |
|  | 3 | 3.189507 | 2.450937 | 3.059579 | 2.79594 |
|  | 4 | 3.138336 | 3.241511 | 3.264058 | 3.138336 |
|  | 5 | 2.543238 | 3.418429 | 3.5801 | 3.434262 |
|  | 6 | 3.317278 | 2.955359 | 3.024437 | 3.324952 |
|  | 7 | 2.706947 | 3.211692 | 2.394957 | 2.907945 |
|  | 8 | 3.819366 | 3.810552 | 2.955359 | 3.498331 |
|  | 9 | 3.332643 | 2.578741 | 3.264058 | 3.379165 |

Table 5 The relative expression of protein of p-MEK1/2 in colon tissue

| Group | Number | p-MEK1/2/GAPDH |
| --- | --- | --- |
| Control group | 1 | 0.066948 |
|  | 2 | 0.049564 |
|  | 3 | 0.029028 |
|  | 4 | 0.098976 |
|  | 5 | 0.035470 |
|  | 6 | 0.059193 |
|  | 7 | 0.058235 |
|  | 8 | 0.037303 |
|  | 9 | 0.086917 |
| Model group | 1 | 0.877839 |
|  | 2 | 0.573067 |
|  | 3 | 0.597369 |
|  | 4 | 0.798832 |
|  | 5 | 0.825265 |
|  | 6 | 0.575180 |
|  | 7 | 0.507530 |
|  | 8 | 0.785196 |
|  | 9 | 0.917283 |
| Positive drug group | 1 | 0.356915 |
|  | 2 | 0.315998 |
|  | 3 | 0.262494 |
|  | 4 | 0.338018 |
|  | 5 | 0.430954 |
|  | 6 | 0.304526 |
|  | 7 | 0.242231 |
|  | 8 | 0.381130 |
|  | 9 | 0.405168 |
| WJP group | 1 | 0.218711 |
|  | 2 | 0.117133 |
|  | 3 | 0.107626 |
|  | 4 | 0.325695 |
|  | 5 | 0.335642 |
|  | 6 | 0.195727 |
|  | 7 | 0.049311 |
|  | 8 | 0.309970 |
|  | 9 | 0.223359 |

Table 6 The relative expression of protein of p-ERK1, p-ERK2 in colon tissue

| Group | Number | p-ERK1 | p-ERK2 |
| --- | --- | --- | --- |
| Control group | 1 | 0.017611 | 0.017815 |
|  | 2 | 0.010836 | 0.012544 |
|  | 3 | 0.017414 | 0.018828 |
|  | 4 | 0.020311 | 0.013674 |
|  | 5 | 0.061083 | 0.035879 |
|  | 6 | 0.035422 | 0.019414 |
|  | 7 | 0.034127 | 0.024638 |
|  | 8 | 0.074140 | 0.029759 |
|  | 9 | 0.179784 | 0.118066 |
| Model group | 1 | 0.509261 | 0.485170 |
|  | 2 | 0.490151 | 0.403067 |
|  | 3 | 0.431842 | 0.328245 |
|  | 4 | 0.522800 | 0.426857 |
|  | 5 | 0.563230 | 0.476449 |
|  | 6 | 0.529170 | 0.448297 |
|  | 7 | 0.414455 | 0.439981 |
|  | 8 | 0.700391 | 0.700539 |
|  | 9 | 0.911535 | 0.759051 |
| Positive drug group | 1 | 0.211882 | 0.137281 |
|  | 2 | 0.088578 | 0.051923 |
|  | 3 | 0.130580 | 0.071797 |
|  | 4 | 0.282434 | 0.190365 |
|  | 5 | 0.269686 | 0.191792 |
|  | 6 | 0.234260 | 0.169324 |
|  | 7 | 0.250950 | 0.189779 |
|  | 8 | 0.352148 | 0.195710 |
|  | 9 | 0.450196 | 0.326149 |
| WJP group | 1 | 0.112938 | 0.060976 |
|  | 2 | 0.020209 | 0.016546 |
|  | 3 | 0.061507 | 0.032201 |
|  | 4 | 0.123144 | 0.065542 |
|  | 5 | 0.101267 | 0.062378 |
|  | 6 | 0.103743 | 0.072540 |
|  | 7 | 0.138987 | 0.078324 |
|  | 8 | 0.207947 | 0.108286 |
|  | 9 | 0.240738 | 0.154649 |

5. The relative expression of mRNA of MEK1, MEK2, ERK1, ERK2 and the relative expression of protein of MEK1/2, p-MEK1/2, ERK1, p-ERK1, ERK2, p-ERK2 in hippocampus tissue in four groups.

Table 7 The relative expression of mRNA of MEK1, MEK2, ERK1, ERK2 in hippocampus tissue

| Group | Number | MEK1 | MEK2 | ERK1 | ERK2 |
| --- | --- | --- | --- | --- | --- |
| Control group | 1 | 1.000000 | 1.000000 | 1.000000 | 1.000000 |
|  | 2 | 0.619854 | 0.733736 | 0.718636 | 0.710382 |
|  | 3 | 1.533328 | 1.372367 | 0.872564 | 0.810378 |
|  | 4 | 1.464086 | 1.130269 | 0.812252 | 1.259921 |
|  | 5 | 1.069300 | 1.225468 | 0.922316 | 1.094294 |
|  | 6 | 1.594753 | 1.035265 | 1.372367 | 1.350350 |
|  | 7 | 1.307369 | 1.457336 | 1.211393 | 1.151355 |
|  | 8 | 0.895025 | 1.477679 | 0.695762 | 1.044877 |
|  | 9 | 1.295342 | 0.878633 | 0.708742 | 0.880666 |
| Model group | 1 | 5.169411 | 5.451551 | 6.204579 | 2.608704 |
|  | 2 | 6.062866 | 3.837056 | 3.490257 | 3.087987 |
|  | 3 | 1.931873 | 2.496661 | 3.003546 | 2.042024 |
|  | 4 | 1.674039 | 2.7384 | 2.260539 | 2.027919 |
|  | 5 | 2.422785 | 6.020987 | 3.555371 | 2.313376 |
|  | 6 | 2.479415 | 5.278032 | 4.584189 | 2.450937 |
|  | 7 | 1.749165 | 2.153475 | 2.08012 | 3.605002 |
|  | 8 | 3.394816 | 3.954056 | 5.01645 | 2.907945 |
|  | 9 | 5.063026 | 5.92439 | 3.723519 | 3.418429 |
| Positive drug group | 1 | 1.753211 | 1.259921 | 1.268684 | 1.089249 |
|  | 2 | 1.685683 | 1.477679 | 1.262835 | 1.122462 |
|  | 3 | 1.870382 | 1.76949 | 1.757267 | 1.378724 |
|  | 4 | 1.42405 | 1.753211 | 1.016305 | 1.228303 |
|  | 5 | 1.725084 | 1.639588 | 1.28937 | 1.41095 |
|  | 6 | 1.70527 | 1.701334 | 1.753211 | 1.219819 |
|  | 7 | 1.460707 | 1.156688 | 1.378724 | 1.337928 |
|  | 8 | 2.297397 | 1.450617 | 1.35035 | 1.262835 |
|  | 9 | 2.013911 | 1.515717 | 1.662476 | 1.565547 |
| WJP group | 1 | 1.470867 | 1.583738 | 1.765406 | 2.229417 |
|  | 2 | 1.733074 | 2.887858 | 2.158456 | 1.883392 |
|  | 3 | 2.276262 | 2.519842 | 2.378414 | 1.689582 |
|  | 4 | 1.94981 | 2.163449 | 1.650992 | 1.745129 |
|  | 5 | 1.681793 | 1.639588 | 2.590685 | 1.870382 |
|  | 6 | 2.394957 | 2.286805 | 1.617015 | 2.265768 |
|  | 7 | 2.208908 | 1.533328 | 2.608704 | 2.109157 |
|  | 8 | 2.046748 | 2.578741 | 2.094588 | 1.70527 |
|  | 9 | 1.632029 | 2.744735 | 1.624505 | 1.963372 |

Table 8 The relative expression of protein of p-MEK1/2 in hippocampus tissue

| Group | Number | p-MEK1/2 |
| --- | --- | --- |
| Control group | 1 | 0.028472 |
|  | 2 | 0.019270 |
|  | 3 | 0.024317 |
|  | 4 | 0.024470 |
|  | 5 | 0.033464 |
|  | 6 | 0.024076 |
|  | 7 | 0.023394 |
|  | 8 | 0.029690 |
|  | 9 | 0.029344 |
| Model group | 1 | 0.649207 |
|  | 2 | 0.484766 |
|  | 3 | 0.451441 |
|  | 4 | 0.481649 |
|  | 5 | 0.396605 |
|  | 6 | 0.587041 |
|  | 7 | 0.457744 |
|  | 8 | 0.550150 |
|  | 9 | 0.794761 |
| Positive drug group | 1 | 0.230720 |
|  | 2 | 0.143011 |
|  | 3 | 0.042877 |
|  | 4 | 0.182416 |
|  | 5 | 0.081971 |
|  | 6 | 0.266751 |
|  | 7 | 0.185274 |
|  | 8 | 0.147818 |
|  | 9 | 0.218013 |
| WJP group | 1 | 0.414355 |
|  | 2 | 0.272825 |
|  | 3 | 0.075545 |
|  | 4 | 0.374806 |
|  | 5 | 0.312114 |
|  | 6 | 0.366901 |
|  | 7 | 0.525404 |
|  | 8 | 0.336185 |
|  | 9 | 0.283861 |

Table 9 The relative expression of protein of p-ERK1, p-ERK2 in hippocamps tissue

| Group | Number | p-ERK1 | p-ERK2 |
| --- | --- | --- | --- |
| Control group | 1 | 0.047739 | 0.057298 |
|  | 2 | 0.018525 | 0.045541 |
|  | 3 | 0.040566 | 0.058241 |
|  | 4 | 0.048241 | 0.071141 |
|  | 5 | 0.028171 | 0.033500 |
|  | 6 | 0.030539 | 0.036128 |
|  | 7 | 0.137434 | 0.147639 |
|  | 8 | 0.194478 | 0.221741 |
|  | 9 | 0.144880 | 0.187306 |
| Model group | 1 | 0.779391 | 0.932859 |
|  | 2 | 0.580111 | 0.705868 |
|  | 3 | 0.736517 | 0.866325 |
|  | 4 | 0.768346 | 0.821788 |
|  | 5 | 0.873912 | 0.909373 |
|  | 6 | 0.894083 | 0.995456 |
|  | 7 | 0.888845 | 0.930760 |
|  | 8 | 0.738311 | 0.785180 |
|  | 9 | 1.113051 | 1.104496 |
| Positive drug group | 1 | 0.274645 | 0.415302 |
|  | 2 | 0.269017 | 0.345356 |
|  | 3 | 0.155773 | 0.205217 |
|  | 4 | 0.287711 | 0.343715 |
|  | 5 | 0.289986 | 0.326028 |
|  | 6 | 0.561577 | 0.671780 |
|  | 7 | 0.435147 | 0.454339 |
|  | 8 | 0.296957 | 0.363695 |
|  | 9 | 0.575517 | 0.643913 |
| WJP group | 1 | 0.545096 | 0.691632 |
|  | 2 | 0.477852 | 0.597231 |
|  | 3 | 0.341882 | 0.382500 |
|  | 4 | 0.593442 | 0.678472 |
|  | 5 | 0.508613 | 0.575411 |
|  | 6 | 0.738704 | 0.828855 |
|  | 7 | 0.710945 | 0.707807 |
|  | 8 | 0.336204 | 0.387017 |
|  | 9 | 0.857946 | 0.890753 |

5. The correlation between the AWR score at each pressure level and the relative expression of p-ERK1 and p-ERK2.

Table 10 The Spearman Correlation analysis between the AWR score and the relative expression of p-ERK1 and p-ERK2 in the colon tissue

|  |  | AWR score  (20mmHg) | AWR score  (40mmHg) | AWR score  (60mmHg) | AWR score  (80mmHg) |
| --- | --- | --- | --- | --- | --- |
| p-ERK1 | Correlation Coefficient | 0.639^**^ | 0.721^**^ | 0.623^**^ | 0.698^**^ |
|  | P value | ＜0.0001 | ＜0.0001 | ＜0.0001 | ＜0.0001 |
| p-ERK2 | Correlation Coefficient | 0.656^**^ | 0.736^**^ | 0.633^**^ | 0.718^**^ |
|  | P value | ＜0.0001 | ＜0.0001 | ＜0.0001 | ＜0.0001 |
| * *p*<0.05 ** *p*<0.01 | | | | | |
|  |  |  |  |  |  |

|  |  | AWR score  (20mmHg) | AWR score  (40mmHg) | AWR score  (60mmHg) | AWR score  (80mmHg) |
| --- | --- | --- | --- | --- | --- |
| p-ERK1 | Correlation Coefficient | 0.453^**^ | 0.538^**^ | 0.486^**^ | 0.443^**^ |
|  | P value | 0.006 | 0.001 | 0.003 | 0.007 |
| p-ERK2 | Correlation Coefficient | 0.485^**^ | 0.554^**^ | 0.490^**^ | 0.446^**^ |
|  | P value | 0.003 | ＜0.0001 | 0.002 | 0.006 |

Table 11 The Spearman Correlation analysis between the AWR score and the relative expression of p-ERK1 and p-ERK2 in the hippocampus tissue

* *p*<0.05 ** *p*<0.01
